# Recommendations for a paradigm shift in approach to increase the recognition and treatment of sialorrhea in Parkinson’s disease

**DOI:** 10.1016/j.prdoa.2023.100223

**Published:** 2023-10-11

**Authors:** Bruno Bergmans, Veronica Clark, Stuart H. Isaacson, Tobias Bäumer

**Affiliations:** a Department of Neurology, AZ St-Jan Brugge-Oostende AV, Campus Brugge, 8000 Bruges, Belgium; b Department of Neurology, Ghent University Hospital, 9000 Ghent, Belgium; c Independent Researcher, Malta Parkinson’s, PO Box 17, Marsa MTP 1001, Malta; d Private Practice, UK; e Parkinson’s Disease and Movement Disorders Center of Boca Raton, 951 NW 13th Street, Bldg. 5-E, Boca Raton, FL 33486, USA; f Institute of Systems Motor Science, University of Lübeck, CBBM (Building 66), Ratzeburger Allee 160, 23562 Lübeck, Germany

**Keywords:** Sialorrhea, Parkinson’s disease, Botulinum toxin, BoNT, Recommendation

## Abstract

Sialorrhea, or drooling, is defined as excessive saliva accumulation and unwanted loss of saliva from the mouth or over the tongue and into the pharynx. It constitutes one of the most frequent and bothersome complaints of patients with Parkinson’s disease (PD), affecting up to 84% of them. Sialorrhea is a distressing and challenging condition that may result in social isolation, embarrassment, depression, skin infections, poor oral health, and aspiration pneumonia. To better understand the burden of sialorrhea on patients with PD, Parkinson’s Europe carried out a worldwide patient survey which showed that sialorrhea remains an underrecognized and undertreated issue in patients with PD. This is especially problematic because effective therapeutic options are available. This article presents the results of the Parkinson’s Europe Sialorrhea Survey, which were considered by a multidisciplinary panel of experts to provide recommendations for improving the awareness, diagnosis, management, and treatment of sialorrhea in patients with PD. A shift in the treatment paradigm for sialorrhea in patients with PD is emerging. It is essential to better educate patients, family members, caregivers, and healthcare professionals about sialorrhea; to engage all those involved to actively discuss sialorrhea and measure its impact on quality of life; and to recognize the role of botulinum toxin and speech and language therapy as first-line therapies.

## Highlights

• Sialorrhea is a common and debilitating consequence of Parkinson’s disease.
• Sialorrhea is underrecognized and undertreated in patients with Parkinson’s disease.
• Recommendations are made to improve care and reduce overall impact of sialorrhea.
• Better education of patients, family, caregivers, and healthcare team is essential.
• Patient-centered treatment combines botulinum toxin with speech/language therapy.

## 1 Introduction

Sialorrhea refers to excessive saliva accumulation and the unintentional loss of saliva from the mouth (anterior sialorrhea), or less frequently over the tongue and into the pharynx (posterior sialorrhea) [1]. It is a common non-motor problem experienced by patients with Parkinson’s Disease (PD) and those with other chronic neurological conditions [1], [2]. Although reported prevalence rates of sialorrhea vary widely across different studies, it can affect up to 84 % of people with PD [3], [4]. Multiple factors are associated with sialorrhea in PD [4], [5]; it increases with patient age as well as duration and severity of PD but can be present at any stage of the disease and at all ages [3], [6].

In healthy adults, most saliva is produced by the submandibular (60 %) and parotid (30 %) glands, with the remaining 10 % being secreted from the sublingual and minor glands [7]. The relative contribution of the different salivary glands varies between the unstimulated and stimulated (e.g., eating, chewing) state, with the amount, flow rate, and composition of saliva being affected [7]. Unstimulated saliva is mostly produced by the submandibular glands and is rich in mucin, while stimulated saliva is secreted predominantly by the parotid glands and is rich in ptyalin and other enzymes [7], [8]. Saliva has numerous functions [9]; it plays an important role in oral/dental health, the first stage of digestion, and supports clear speech [7].

The composition and flow of saliva is altered in PD [7], although the pathophysiology of sialorrhea in PD is not yet fully understood [6]. Mechanisms may include overproduction of saliva (increased velocity of saliva excretion from the parotid gland); poor retention of saliva within the oral cavity caused by dysfunction or weakness of muscles in the mouth, tongue, and throat (hypomimia, involuntary mouth opening, stooped posture or drooping of head); and reduced salivary clearance due to lingual bradykinesia, oropharyngeal dysphagia, and upper esophageal sphincter dysfunction [6], [10]. Some studies have found that cognitive impairment can increase the presence and severity of sialorrhea in PD [6]. Notably, sialorrhea usually occurs during ‘off' periods of symptom control in PD [4]; thus, optimization of dopaminergic therapy may improve motor symptoms including swallowing function [6], [10].

Sialorrhea is rarely considered a major symptom of PD but is often rated as one of the most bothersome and debilitating problems [11]. Patients can experience sialorrhea at night only (nocturnal) or during both the day and night (diurnal) [12]. Sialorrhea can have physical, emotional, and social consequences that impact everyday life. Physical consequences of sialorrhea include perioral chapping, excoriation of the skin around the mouth, oral and dental hygiene problems, swallowing difficulties (dysphagia), speech difficulties (dysarthria), and sleep disturbance [2], [13]. Patients affected by PD and sialorrhea can experience xerostomia (subjective feeling of dry moth) [14]. Pooling of saliva at the back of the throat may also lead to cough and aspiration pneumonia, which increases the risk of mortality [15], [16]. Drooling onto clothing can lead to frequent changes of clothes during the waking day and overnight, and drooling onto the floor can be a fall risk. Common psychosocial effects of sialorrhea include social isolation of people with PD, due to embarrassment and family avoiding physical contact, as well as an increased burden on carers (e.g., washing clothes, restricted social life) [7]. Patients with PD experiencing sialorrhea report a greater impairment in quality of life compared with those without sialorrhea due to stigma and difficulties with communication and activities of daily living [17]. Patients with PD and their caregivers have reported that the non-motor symptoms of PD, including sialorrhea, have a greater impact than motor symptoms on their quality of life [11], [18], [19].

Options for treating sialorrhea in people with PD include non-pharmacological therapy (behavioral treatments, speech and language therapy), pharmacological therapy (oral anticholinergics, botulinum toxin [BoNT] injections into the major salivary glands), or invasive treatments (surgery, radiotherapy) [8], [10]. Current management guidelines for PD, such as the UK National Institute for Health and Care Excellence (NICE) guidelines [20], recommend non-pharmacological techniques (e.g., speech and language therapy) as first-line therapy to control sialorrhea. They state that pharmacological management should only be considered if the non-pharmacological approach has been ineffective [20]. Currently, the recommended order of medications for treating sialorrhea in PD is glycopyrronium bromide, BoNT, then other anticholinergics. Surgery or irradiation of salivary glands are reserved for patients who are unable to tolerate oral medications or BoNT and are rarely used in practice [1], [21]. Guidelines for managing sialorrhea in other countries follow a similar approach [22].

Despite the considerable burden and availability of multiple treatment modalities, sialorrhea in PD remains underrecognized by physicians and, consequently, undertreated [8].

In 2020, Parkinson’s Europe (formerly the European Parkinson’s Disease Association) conducted a worldwide survey to understand the impact of sialorrhea in people with PD and to raise awareness of treatment options [23]. The present review summarizes the recommendations of a multidisciplinary expert panel that were developed using insight gained from the Sialorrhea Survey [23] together with consideration of the scientific literature. The aim of these recommendations is to help promote education of multidisciplinary PD care teams, patients, and their families/carers about sialorrhea and to achieve important changes in clinical practice, leading to improved and earlier recognition and treatment of sialorrhea in people with PD.

## 2 Methods

### 2.1 Sialorrhea survey

The Parkinson’s Europe Sialorrhea Survey was conducted between 14 October 2020 and 30 November 2020 and consisted of an online self-completed questionnaire that was available in English, German, French, and Spanish [23]. Information collected included demographic and clinical data (age, gender, time since PD diagnosis), awareness of sialorrhea as a symptom of PD, severity and frequency of drooling, the burden of sialorrhea from a physical, wellbeing, and daily living perspective for people living with PD, and information about if, when, and how sialorrhea is addressed by healthcare professionals.

### 2.2 Development of expert panel recommendations

A multidisciplinary panel of experts gathered in February 2022 during the Adult Sialorrhea Expert Forum – an event facilitated by Merz Therapeutics GmbH and Parkinson’s Europe – to discuss the Sialorrhea Survey Report and identify current barriers to sialorrhea diagnosis and management. Some of the participants, who are the authors of this paper, formed a working group to further debate, find consensus, and propose recommendations on how to improve these aspects of care and reduce the overall impact of sialorrhea on patients with PD.

## 3 Results of sialorrhea survey

The results of the Sialorrhea Survey have been reported [23]. A total of 382 respondents qualifying as a person with PD or a caregiver of a person with PD completed the survey. They were from Belgium (11 %), Canada (24 %), France (5 %), Germany (8 %), Spain (10 %), the United Kingdom (20 %), the United States (5 %), with the remainder from at least 20 other countries. Of the 382 respondents, 63 % were aged between 60 and 80 years, 60 % were males, 74 % were people with PD while 18 % were either a family member or carer of someone with PD, and 80 % of the patients in the survey had been diagnosed with PD more than 4 years previously. Some respondents (9 %) did not have PD but another related condition (e.g., multiple system atrophy). The majority of respondents (86 %) were aware that sialorrhea is a symptom of PD.

### 3.1 Impact of sialorrhea on patients

**Fig. 1:**
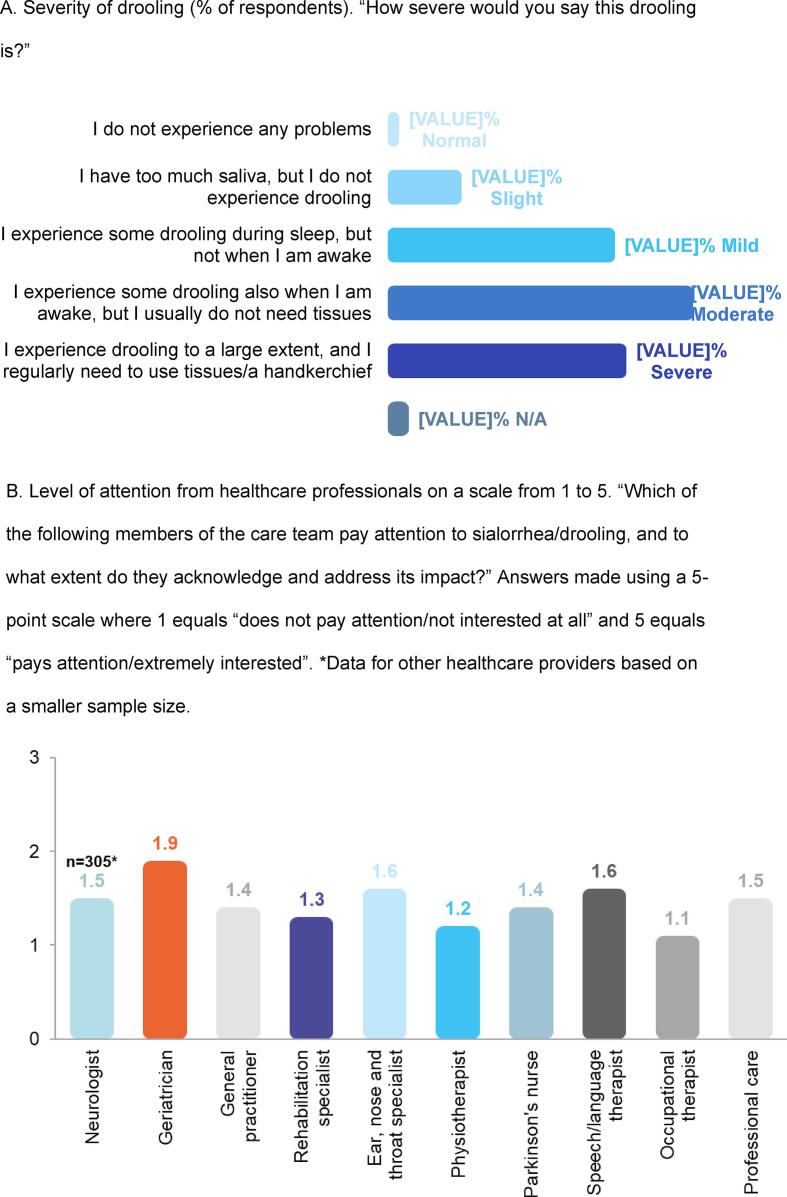
Selected results from the Parkinson’s Europe Sialorrhea Survey [23]. N/A, not available. A. Severity of drooling (% of respondents). “How severe would you say this drooling is?” B. Level of attention from healthcare professionals on a scale from 1 to 5. “Which of the following members of the care team pay attention to sialorrhea/drooling, and to what extent do they acknowledge and address its impact?” Answers made using a 5-point scale where 1 equals “does not pay attention/not interested at all” and 5 equals “pays attention/extremely interested”. *Data for other healthcare providers based on a smaller sample size.

In total, 88 % of respondents reported that they experienced sialorrhea, and the majority felt they had moderate (35 %) or severe (27 %) drooling, defined as some drooling during daytime or excessive drooling during daytime requiring the use of tissues, respectively (Fig. 1A). The survey question on the frequency of drooling revealed that sialorrhea was “frequently” or “constantly” present in 63 % of respondents. More than 70 % of respondents reported having problems with swallowing and 56 % experienced dry mouth at times.

For more than one third of patients, sialorrhea caused a significant impact on key aspects of their life. Sialorrhea was troublesome for many reasons: respondents frequently reported social embarrassment (40 %), impact on speech (21 %), physical discomfort (15 %), and impact on eating and swallowing (13 %) as key issues, whereas skin irritation (3 %) was the key issue for only a few people. The impact of sialorrhea on patients’ daily and social lives was as severe as the impact on their physical health (on the respiratory tract, skin irritation), and patients’ self-esteem was particularly affected.

### 3.2 Healthcare provider attention to and treatment of sialorrhea

Despite the considerable impact of sialorrhea on the wellbeing of patients with PD, the Sialorrhea Survey Report revealed that sialorrhea is often neglected by patients and their care team [23]. Almost half of the respondents (45 %) had never talked about drooling with any member of their healthcare team. While many healthcare professionals from several different disciplines are seeing patients with PD and sialorrhea, patients felt that this symptom and its impact was neglected by all members of the care team. When asked about the level of attention that sialorrhea was getting from the healthcare professionals involved in their care, patients rated all professional categories below 2 on a 5-point scale (Fig. 1B). Of even greater concern is that only one quarter (27 %) of patients received a medical diagnosis of sialorrhea and less than half of respondents (44 %) were ever recommended any therapy. The most common treatments provided were speech and language therapy followed by simple swallow reminders or other drugs/medication/aids such as chewing gum. In general, patients were not very satisfied with the result of these therapies and felt there was room for improvement.

Together, these results suggest a lack of awareness of sialorrhea, its impact on patients’ lives, and the treatment options available to achieve successful symptom management.

## 4 Potential barriers to diagnosis and management of sialorrhea

Based on the results of the Sialorrhea Survey and their clinical experience/knowledge, the multidisciplinary panel of experts identified several barriers to proper diagnosis and management of sialorrhea in PD, which could be separated into patient-level and healthcare professional-level factors (Table 1).

**Table 1:** Barriers to diagnosis and management of sialorrhea in people with Parkinson’s disease (PD).

**Barrier identified**	**Explanation**
**Patient-level factors**	
Embarrassment	The patient is ashamed or afraid to talk about their sialorrhea problems
Lack of awareness	The patient does not link their non-motor symptoms with PD or is not aware that treatments for sialorrhea are available
Relative perception of symptoms	The patient has several concurrent motor and non-motor symptoms and does not perceive sialorrhea as an important issue that requires medical attention or forgets it amongst their other symptoms.
Cognitive decline	The patient is unable to discuss symptoms with their physician due to cognitive decline and requires the involvement of a caregiver
**Healthcare profession level factors**	
Low level of attention	Healthcare professionals may not ask patients if they are experiencing problems with sialorrhea, or with their speech and swallowing, which are often associated with sialorrhea
Low recognition of impact	Healthcare professionals may ignore non-motor symptoms and focus only on motor problems, or they may identify sialorrhea but fail to address it due to a lack of recognition of its impact on the patient’s life [25]
Lack of treatment awareness	Healthcare professionals may not be informed about appropriate treatment options for sialorrhea and feel that there is not much that can be done; are only aware of antimuscarinic drugs to treat sialorrhea, which come with multiple adverse events; or are unaware of or have misconceptions about botulinum toxin therapy
Lack of disease education	Speech and language therapists may not be specialized in PD or may not be aware of treatment options and rehabilitation techniques to help patients control their saliva
Lack of treatment availability	Access to botulinum toxin therapy and/or speech and language therapy may be challenging in some regions due to a lack of trained healthcare professionals and country-specific reimbursement challenges

## 5 Expert panel recommendations for optimal management of sialorrhea in PD

### 5.1 Holistic multidisciplinary care

**Fig. 2:**
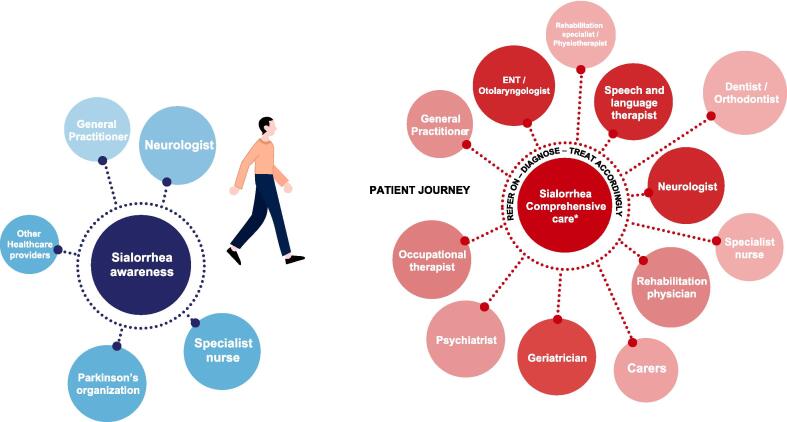
Patient-centered, multidisciplinary team approach to management of sialorrhea in Parkinson’s disease patients. *Specific to each country. ENT, ear, nose, throat.

The expert panel highlighted the importance of developing a holistic and patient-centered approach to the management of sialorrhea in PD that educates and empowers patients and caregivers to talk to their healthcare team about drooling and its impact on them. A multidisciplinary team approach (Fig. 2) is essential to adequately address all the patient's needs. The organization of the multidisciplinary care teams will vary by country and how care is provided between different centers. It is recommended that patients have a main point of contact to coordinate care between the patient, caregivers, and other members of the multidisciplinary team. Treatment should be overseen by the neurologist or even a geriatrician who specializes in PD. The goal is to establish a network of specialists (e.g., psychiatrists, geriatricians, speech therapists, etc.) with different expertise who can work together so that patients receive optimal management of their PD [24].

### 5.2 Paradigm shift in the management of sialorrhea in patients with PD

**Fig. 3:**
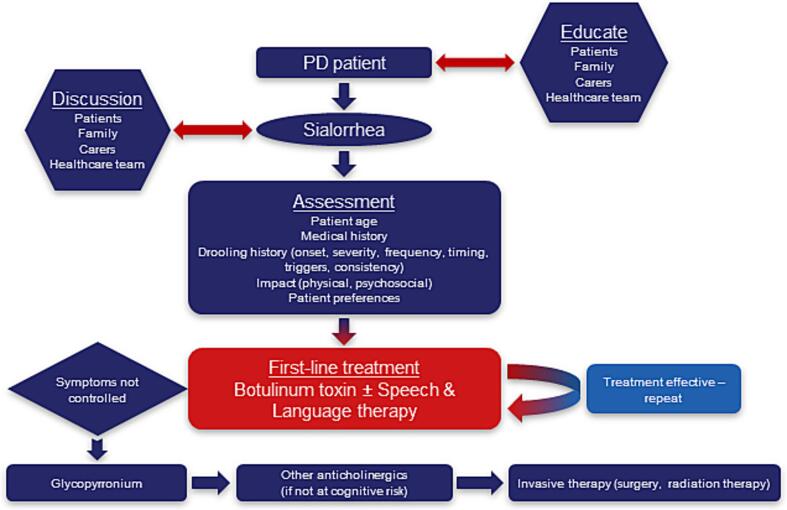
Recommended paradigm shift for the management of sialorrhea in patients with Parkinson’s disease (PD).

The expert panel has proposed a paradigm shift in the management of sialorrhea in patients with PD (Fig. 3). Specifically, they recommend: 1) Increased awareness and education of sialorrhea as a non-motor symptom of PD to facilitate an earlier diagnosis; 2) Speech and language therapy and/or BoNT as first-line therapies, and 3) Cautious use of anticholinergic treatments.

#### 5.2.1 Earlier diagnosis of sialorrhea

For a clinical diagnosis of sialorrhea, it is important to collect information on patient age, medical history (mental status, neurologic symptoms and preexisting conditions, previous pulmonary infections, current medications), and history of drooling, including onset, duration, timing or trigger, severity/amount, consistency (thin, watery, or viscous), previous treatments and response, and the physical and psychosocial impact of drooling on patient and caregiver [26]. Physical examination should include evaluating the patient’s level of alertness, emotional state, nutrition and hydration status, and head posture. The oral cavity should be examined for the following: poor lip seal; issues with teeth, gums, tonsils, and tongue; abscesses; intraoral sensitivity; swallowing; anatomical abnormalities (nasal blockage, malocclusion, and jaw stability); and visible saliva (hypersecretion, viscosity) [26]. Severity of sialorrhea can be assessed using various methods that range from asking about the number of bibs/tissues used daily or the frequency of changing clothes, through use of subjective rating scales of the frequency and severity of drooling, to objective measures that quantify the amount of saliva produced [27].

Specific actions for achieving an earlier diagnosis of sialorrhea recommended by the expert panel are summarized in Table 2.

**Table 2:** Expert panel recommendations for the management of sialorrhea in Parkinson’s disease (PD).

**Theme**	**Specific actions**
Earlier diagnosis of sialorrhea	• Raise awareness of sialorrhea among patients with PD, primary and secondary care physicians, and the extensive multidisciplinary team to better engage patients, family members, and caregivers to discuss sialorrhea and properly identify its impact on their lives. Educate all healthcare professionals to better identify sialorrhea symptoms and to make onward referrals as required. Involve caregivers and family members in physician visits to facilitate conversations about sialorrhea. This is particularly important when the patient with PD presents with communication or cognitive difficulties. Use an exhaustive list of non-motor symptoms with patients at every physician visit to ensure that lesser-known symptoms, such as sialorrhea, are not overlooked. Ask specific questions and give examples of possible day-to-day issues to support the conversation around sialorrhea, e.g., do you ever experience any excess saliva in your mouth? Do you ever experience wet lips or drooling of saliva? Do you ever have a wet voice after you eat and drink? Do you feel the need to cough or clear your throat due to excess saliva in your throat? Do you experience stains on your T-shirt? Do you experience a risk of falling due to saliva on the floor? Evaluate the impact of sialorrhea on the patient’s quality of life rather than relying solely on assessments of the volume, frequency, or severity of drooling; some patients may find mild sialorrhea really bothersome, whereas others may not. Consider using a standard rating scale, such as the Drooling Severity and Frequency Scale (DSFS) (Table 3) [27], [28], to quickly assess sialorrhea, identify patients early on, and to monitor changes over time. **Table 3:** Drooling Severity and Frequency Scale (DSFS) [28]. **Drooling** **Points** Severity Dry – never drools Mild – only lips wet Moderate – drool reaches the lips and chin Severe – drool drips off chin and onto clothing Profuse – drooling off the body and onto objects (furniture, books) Frequency No drooling Occasionally drools Frequently drools Constant drooling 1 2 3 4 5 1 2 3 4 The score of the DSFS equals the sum of the severity and frequency sub-scores. Educate healthcare professionals about the importance of a multidisciplinary team approach.
First-line therapy	• Educate patients adequately about sialorrhea and its treatment. Refer patients to a speech and language therapist early in the disease course. Educate clinicians adequately about sialorrhea recognition and treatment with botulinum toxin and increase the number of clinicians who can give botulinum toxin injections.
Cautious use of anticholinergics	• Avoid anticholinergic drugs when possible due to their systemic side effects. If an anticholinergic drug needs to be used, use one that has minimal entry to the central nervous system (CNS), such as glycopyrronium bromide.

#### 5.2.2 First-line therapies

The goal of sialorrhea treatment is to reduce excessive salivation while maintaining a moist and healthy oral cavity and preventing xerostomia [26].

The expert panel recommends speech and language therapy and/or BoNT as first-line therapies (see Table 2 for specific actions).

Speech and language therapy is one of many non-pharmacological options available for the treatment of sialorrhea, some of which have not demonstrated efficacy in clinical trials [1]. Speech and language therapy aims to maximize and rehabilitate the swallowing function. It can improve jaw stability and closure; increase tongue mobility, strength, and positioning; improve lip closure (especially during swallowing); and decrease nasal regurgitation during swallowing. Evidence-based therapy programs such as the Lee Silverman Voice Treatment can improve both the patient’s voice and swallowing [29].

BoNT treatment should be considered a first-line therapy for sialorrhea in patients with PD by all healthcare professionals involved in their treatment because of its proven effectiveness in reducing the frequency and severity of drooling, good tolerability, and simple injection technique [30], [31]. Two BoNT preparations have regulatory approval for the treatment of chronic sialorrhea in adults based on data from large randomized controlled trials: rimabotulinumtoxinB (serotype B) is approved by the US Food and Drug Administration (FDA) [32], and incobotulinumtoxinA (serotype A) is approved by both the European Medicines Agency (EMA) and the FDA [1], [8], [33], [34].

When injected into the salivary glands, BoNT selectively binds to muscarinic cholinergic nerve terminals and temporarily inhibits the release of acetylcholine, reducing salivary secretions [21]. The advantages of BoNT injections include a reduced frequency of administration versus anticholinergic medications which must be taken multiple times/day; a quick and minimally invasive treatment – dosing and injection technique are straightforward using anatomical and/or ultrasound guidance [22], [35]; treatment benefits can be seen as early as 1 week after injection and last for 3–6 months [36]. Adverse effects related to BoNT injections into salivary glands may include pain at the injection site, dry mouth, and dysphagia, but are uncommon and temporary.

**Fig. 4:**
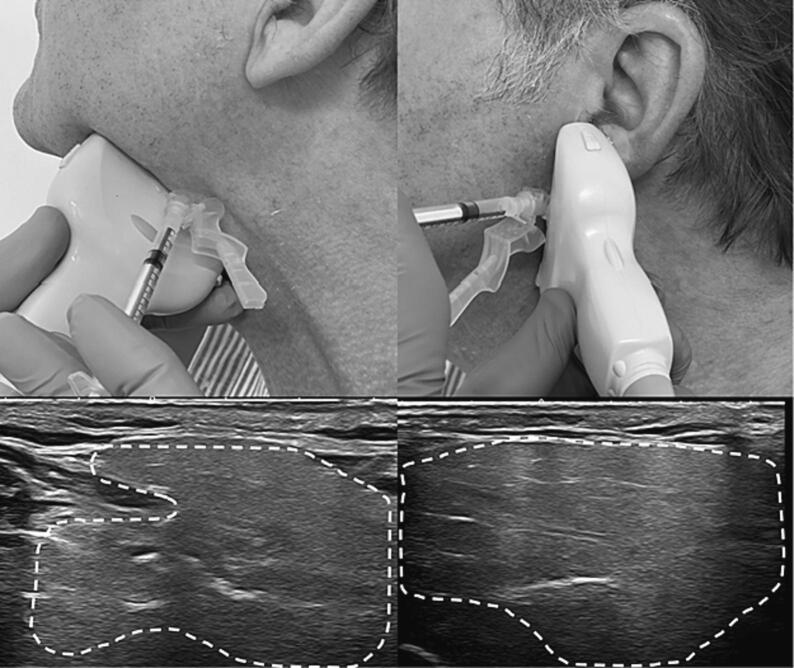
Illustration of the ultrasound-guided injection. The probe positions and corresponding ultrasound images are shown for the submandibular gland on the left and the parotid gland on the right. The ultrasound probe (14 MHz linear transducer) is positioned so that the gland to be injected is displayed with its largest portion in the center of the image. The needle is inserted on the long side of the probe. See accompanying videos 1 and 2 in the Supplementary material.

BoNT is injected into either the parotid or submandibular glands or both glands. Combined injection of the submandibular and parotid glands is typically necessary to reduce both resting (mainly submandibular) and stimulated (mainly parotid) saliva secretion [22]. Although anatomical landmarks can be used to locate the salivary glands [2], [4], [37], precise delivery and a better safety profile is achieved when BoNT injections are made under ultrasound guidance [31], [35], [38]. The injections should be performed by someone specifically trained to do them. The injection procedure can be performed on an outpatient basis and does not require anesthesia. Fig. 4 and the accompanying videos (Supplementary material; videos 1 and 2) show how to locate and administer BoNT injections into the parotid and submandibular glands. For patients with suboptimal results using this injection approach, there is evidence to use a slightly different ultrasound-guided approach [35], [39] which is also demonstrated in the Supplementary material (video 3).

Currently, the recommended total dose of incobotulinumtoxinA is 100 U: injected as 30 units in each parotid gland and 20 units in each submandibular gland. For patients with dysphagia, it is more cautious to start with 75 U. The recommended total dose of rimabotulinumtoxinB is 1500–3500 U, administered as 500–1500 U per parotid gland and 250 U per submandibular gland [36].

Only a few side-effects of BoNT have been reported (3–5 % dry mouth and 0–3 % dysphagia). Although the oral health of people with PD is worse than that of healthy individuals [40], BoNT injections may increase the risk of dental caries and other adverse oral health effects by decreasing salivary flow and changing the composition of saliva [41], [42]. Oral health care, dental hygiene and regular dental appointments are recommended for preventing these problems.

#### 5.2.3 Anticholinergic medication use

The expert panel recommends cautious use of anticholinergics in the treatment of sialorrhea in people with PD (Table 2).

Anticholinergics such as hyoscine hydrobromide, atropine, and scopolamine have been used to manage sialorrhea [2], but systemic and/or central nervous system (CNS) adverse effects limit their usefulness. Evidence of the effectiveness of anticholinergic drugs to treat sialorrhea is limited and significant caution is required when prescribing anticholinergics to patients with PD. Because anticholinergics are not specific to the muscarinic receptors of the salivary glands, patients using such medication for sialorrhea management are at risk of systemic adverse effects including urinary retention, gastrointestinal issues (e.g., constipation), increased intraocular pressure, cessation of perspiration, hyposalivation, xerostomia, increased body temperature, and blurred vision [2]. Moreover, many anticholinergics enter the CNS and may increase the risk of cognitive impairment [43], [44] and dementia in patients with PD [45], [46]. Also, polypharmacy may increase the anticholinergic burden and lead to intolerable adverse effects, especially in elderly patients.

Glycopyrronium bromide is an anticholinergic drug that does not cross the blood–brain barrier and can be considered for the management of sialorrhea in some patients with PD [47].

## 6 Conclusions and future directions

The results of the Parkinson’s Europe Sialorrhea Survey formed the basis for expert panel discussions to develop recommendations for improving the early recognition and management of sialorrhea in patients with PD. The survey revealed that despite being a common symptom of PD, sialorrhea is often underrecognized and undertreated. As effective and safe treatments are available, the expert panel considers that a paradigm shift in the management of sialorrhea is needed. The first step should be to raise awareness and provide education about sialorrhea in PD to all patients, families, carers and healthcare professionals involved in the care of such patients, to improve earlier recognition and diagnosis. For patients whose quality of life is affected by sialorrhea, a multidisciplinary team of healthcare practitioners should take a holistic and patient-centered approach to treatment, which should involve a combination of BoNT and speech and language therapy as first-line therapies. BoNT injections can be administered using a simple and minimally invasive technique, and are an effective, and well tolerated treatment that has fewer side effects than anticholinergics.

Further high-quality studies are needed to determine the optimal treatment of sialorrhea in PD and which combination of approaches works best.

## Funding sources and conflicts of interest

The authors received no specific funding for this article. The Adult Sialorrhea Expert Forum, the writing of this publication and the submission fees were funded by Merz Therapeutics GmbH.

## 8 Ethical compliance statement

This work did not require the approval of an institutional review board or informed patient consent. The authors confirm that they have read the Journal’s position on issues involved in ethical publication and affirm that this work is consistent with those guidelines.

## Declaration of interest

BB has a clinical practice at AZ St-Jan Brugge-Oostende AV in Bruges, Belgium, and is an academic consultant at Ghent University Hospital, Ghent, Belgium. He has served as an advisory board member for Allergan, Merz, AbbVie, and UCB and as a speaker for Zambon, Merz, and AbbVie. VC has an independent SLT practice in the UK and is an employee of NHS England. She has received speaker fees from Merz. SI has received honoraria for research, consulting, CME, and/or speaker bureau activities from Abbvie, Ipsen, Merz, Revance, and Supernus. TB is an employee of the University Hospital Schleswig Holstein. He has received funding from the German Research Foundation (DFG, BA 6375/2-1); speaker and consultant fees from Pelzerhaken Children's Centre, Allergan/AbbVie, Ipsen, and Merz; research funding from Allergan/AbbVie, Ipsen, and Merz; and was supported with exhibition ultrasound equipment on loan from Canon and Esaote.

## CRediT authorship contribution statement

**Bruno Bergmans:** Conceptualization, Writing – review & editing. **Veronica Clark:** Conceptualization, Writing – review & editing. **Stuart H. Isaacson:** Conceptualization, Writing – review & editing. **Tobias Bäumer:** Conceptualization, Writing – review & editing.

## Declaration of Competing Interest

The authors declare that they have no known competing financial interests or personal relationships that could have appeared to influence the work reported in this paper.
