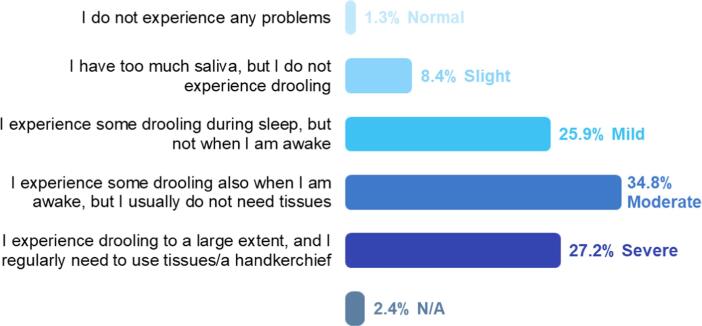# Corrigendum to “Recommendations for a paradigm shift in approach to increase the recognition and treatment of sialorrhea in Parkinson’s disease” [Clin. Parkinsonism Related Dis. 9 (2023) 100223]

**DOI:** 10.1016/j.prdoa.2024.100250

**Published:** 2024-04-18

**Authors:** Bruno Bergmans, Veronica Clark, Stuart H. Isaacson, Tobias Bäumer

**Affiliations:** a Department of Neurology, AZ St-Jan Brugge-Oostende AV, Campus Brugge, 8000 Bruges, Belgium; b Department of Neurology, Ghent University Hospital, 9000 Ghent, Belgium; c Independent Researcher, Malta Parkinson’s, PO Box 17, Marsa MTP 1001, Malta; d Private Practice, UK; e Parkinson's Disease and Movement Disorders Center of Boca Raton, 951 NW 13th Street, Bldg. 5-E, Boca Raton, FL 33486, USA; f Institute of Systems Motor Science, University of Lübeck, CBBM (Building 66), Ratzeburger Allee 160, 23562 Lübeck, Germany

The authors regret that a formatting error was present in Figure 1A, which has been corrected in the corrigendum. The revised figure is shown below.

The authors would like to apologise for any inconvenience caused.

Fig. 1. Selected results from the Parkinson’s Europe Sialorrhea Survey [23]. N/A, not available. A. Severity of drooling (% of respondents). “How severe would you say this drooling is?”.

**Figure:**